# A New Contact Structure and Dielectric Recovery Characteristics of the Fast DC Current-Limiting Circuit Breaker

**DOI:** 10.3390/s25051538

**Published:** 2025-03-01

**Authors:** Zhiyong Lv, Xiangjun Wang, Jinwu Zhuang, Zhuangxian Jiang, Zhifang Yuan, Jin Wu, Luhui Liu

**Affiliations:** 1 College of Electrical Engineering, Naval University of Engineering, Wuhan 430000, China; 2 College of Electrical Engineering, Zhejiang University, Hangzhou 310058, China; 3 National Key Laboratory of Science and Technology on Vessel Integrated Power System, Naval University of Engineering, Wuhan 430000, China

**Keywords:** FDCCLCB, high rising rate fault current, dielectric recovery, parallel multi-point contacts, multi-point arcing

## Abstract

In order to solve the problem of slow dielectric recovery caused by large arc energy when interrupting a high rising rate fault current in a fast DC current-limiting circuit breaker (FDCCLCB), a new contact structure with multi-point static contacts in parallel is proposed. Based on the principle of parallel multi-point contacts, the new structure can form the arc mode during multi-point arcing when the contacts are separated, reduce the arc energy of each finger contact, effectively reduce the ablation effect of the arc on the contact, and improve dielectric recovery ability after the arcing of the contact. Using high-speed camera technology to photograph the arc shape of the new contact, the assumption of multi-point arcing is verified, and a dielectric recovery experimental platform is built to study the dielectric recovery characteristics of the new contact structure. The experimental results show that, when the arc energy is 3.6 J and the dielectric recovery time is 60 µs, the critical field strength reaches 1.5 V/µm; when the arc energy is increased to 22 J, the critical field strength is 0.6 V/µm under the same dielectric recovery time. It can be seen that reducing the arc energy of the contact can effectively improve the dielectric recovery ability of the contact. Due to the magnetic field coupling between each finger contact, the current and arc energy on each contact are different, resulting in a weak point of breakdown and finger contacts at two ends. Finally, in order to solve the problem of large contact current at two ends, a solution to adjust the spacing among contacts is proposed. A genetic algorithm is used to optimize the spacing parameters. The optimization results show that the maximum arc energy of the finger contact is only 19.07% of the total arc energy, which greatly reduces the arc energy of the contact and improves the post-arc recovery ability of the contact.

## 1. Introduction

The marine DC power system has characteristics of short line, low impedance, and small time constant, and the initial rising rate of the short-circuit current usually exceeds 20 A/µs. The short-circuit current can rise to more than 100 kA in a few milliseconds, which suggests high requirements for rapidity of a DC circuit breaker [1,2,3,4].

Fast DC current-limiting circuit breakers (FDCCLCBs) can be mainly divided into the mechanical DC circuit breaker, solid-state DC circuit breaker, and hybrid DC circuit breaker [5]. The solid-state DC circuit breaker and hybrid DC circuit breaker use a power electronic switch to interrupt the current in the main circuit, which leads to high cost, large on-state loss, and poor economy in cases of high current [6,7]. The mechanical DC circuit breaker forms an artificial zero-current point through the commutation circuit, thus completing the DC current by using the AC interrupting principle; the principle is simple, is composed of the mature interrupting unit, capacitor, inductor, and other devices, with outstanding advantages [8,9,10,11]. Researchers have proposed many topology schemes for mechanical DC circuit breakers.

In [12,13], Hasan et al. designed a new bridge topology for short-circuit protection with rated voltage and a current of 230 V/400 A and an expected short-circuit current peak of 75 kA, which limits the short-circuit current peak to 9.3 kA and the recovery voltage peak to 560 V. In [14], Wang et al. designed a new type of DC vacuum current-limiting circuit breaker for the DC 1 kV/400 A, with an expected short-circuit current of 20 kA and initial current rising rate of 5 A/μs, which can limit the peak short-circuit current to 2.5 kA or less. In [15], the Center for Advanced Power Systems (CAPS) in America and the Swiss Federal Institute of Technology jointly developed a current-limiting circuit breaker for a medium voltage DC system for rated voltage, with a current of 12 kV/2 kA and an expected peak short-circuit current of 20 kA, which can limit the peak short-circuit current to 10 kA. In [16], in order to solve the protection problem of a medium voltage DC power system with rated voltage and a current of 5 kV/5 kA, initial current rising rate of 20 A/µs, and expected short-circuit current of 100 kA, Liu et al. proposed a new current-limiting circuit breaker scheme combining the natural commutation and forced commutation principle, which can limit the short-circuit current within 40 kA. In [17], Jia et al. proposed a novel MVDC commutation-interrupting topology that combines a load-carrying branch and an arcing branch in parallel. In contrast to the conventional structure based on semiconductor devices, each branch in the proposed topology contains a mechanical contact, which provides a lower on-state loss and higher voltage-interrupting capacity. In [18], Huo et al. dealt with the principle of AC/DC interruption for low-voltage air circuit breakers. When a fault occurred, by squeezing the arc into constricted insulating slits, the arc resistance was elevated, resulting in an arc voltage that was higher than the system voltage. Hence, the fault current would decrease until the arc plasma could not be maintained. This method can quickly establish a higher voltage than the conventional air arc. In [19], Pei et al. proposed a new and fast DC circuit breaker utilizing a series-connected coupled inductor, which enabled automatic current commutation from the mechanical switch to the semiconductor switch during a fault.

The common features of the above schemes are as follows: in order to achieve the purpose of fast current-limiting, high-speed drive technology is needed to quickly form the opening distance, with fast commutation technology and fast dielectric recovery technology to realize rapid recovery of the mechanical contact after arcing in order to withstand the overvoltage. The key to successful interruption lies in the post-arc dielectric recovery ability of the contact, which is related to the arc energy, the contact speed and the arc shape, etc. [20].

In [21], the dielectric recovery characteristics of the FDCCLCB are studied, and it is concluded that reducing arc energy and increasing the movement speed of contact can improve the critical breakdown voltage of the vacuum interrupter; it was found that the critical breakdown voltage of the vacuum interrupter firstly decreases and then increases with the increase in arcing time. In reference [22], by introducing the voltage ratio coefficient of the electrode, strong evidence demonstrated that the higher arc energy could prolong the effect of electrode temperature on the recovery of post-arc dielectric strength and that the recovery speed of the post-arc dielectric strength decreases as the arc energy increases. In [23], the relationship between dielectric recovery and arcing time under different arc energies and contact separation times, is presented. On the one hand, a longer arcing time leads to larger contact separation, which has a positive effect on dielectric recovery; on the other hand, a longer arcing time results in higher arc energy, which has a negative influence on dielectric recovery. In [24], a longer arc duration increases the surface erosion of the contacts and prolongs the protrusions, due to the arc energy, thereby affecting the dielectric strength of the air gap between the contacts. In addition to reducing the arc energy and increasing the contact speed, the arc shape characteristics also have a great impact on the contact interruption performance. The study of arc shape is helpful in improving the interrupting ability of the contact. In [25], Schulman and Slade used a high-speed camera to photograph the arc shape of Cu–Cr butt contacts, breaking current within the range of 0~11 kA. It was found that one or two initial bridge column arcs always formed after the rupture of the molten metal bridge. The arc development can be divided into three sequences, based on the peak current and the average separation current per bridge column. In [26], the shape characteristics of the arc at the small gap are photographed by high-speed camera technology. In the experiment, a flat contact is used; the current is 3 kA, and the contact opening distance is less than 1 mm. The results show that, in the case of a small gap, the arcing time is short, and the arc has no time to change into a diffusion arc, showing the shape characteristics of a single column or double bridge column arc. In [20], the arc shape of the fast DC air circuit breaker is photographed by high-speed camera technology, and it is concluded that whether the circuit breaker can be interrupted successfully is related to the arc shape of the single column and double column. In [27], in order to solve the problem of fault current interruption with a high rising rate in a low-voltage marine DC power system, a DC current-limiting circuit breaker based on forced commutation principle is proposed. After the experiment, it was found that this type of circuit breaker has the problem of contact ablation caused by large arc energy, and an approximate non-arc control strategy is proposed to solve this problem. Although the new arcless breaking strategy can reduce the arc energy and reduce the contact ablation, it is necessary to carry out forced commutation before opening the contact, which will cause the loss of capacitive energy from the diode branch.

In order to reduce the arc energy of the contact and improve the post-arc dielectric recovery speed of the contact, a new contact structure with parallel multi-point static contacts is proposed. Based on the principle of the parallel multi-point static contacts, the new structure can form an arc mode of multi-point arcing when the contact is separated, effectively reduce the arc energy of the finger contact point, and greatly improve the post-arc dielectric recovery ability of the contact. The arc shape of the parallel multi-point static contacts is photographed using a high-speed camera, and the assumption of multi-point arcing is verified; the dielectric recovery experimental platform is built to study the dielectric recovery characteristics of the new contact structure. The results show that the arc energy has an important influence on the dielectric recovery of the contact. The arc energy is dispersed to each contact by the parallel multi-point contact method, which avoids the existence of weak breakdown points and improves the post-arc dielectric recovery ability of the contact. Finally, in order to solve the problem of large contact current at two ends of the contact, a genetic algorithm is used to optimize the spacing among each contact. The optimization results show that the maximum current of the finger contact is only 19.07% of the total current, which greatly reduces the arc energy of the contact and improves the dielectric recovery ability of the contact.

## 2. Topology and Working Principle of the FDCCLCB

**Figure 1:**
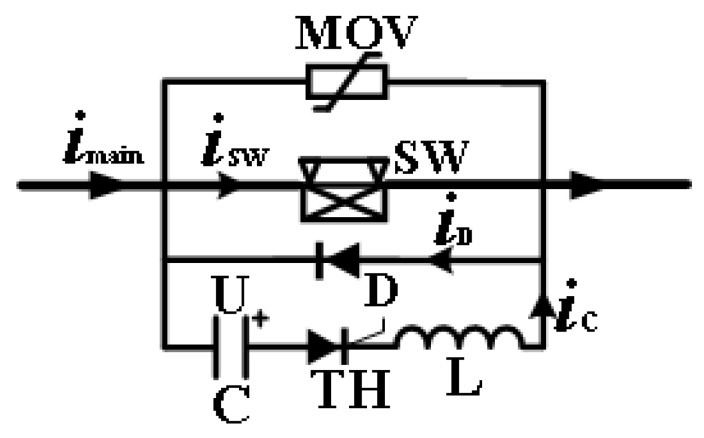
Topology diagram of the FDCCLCB.

The FDCCLCB is composed of a high-speed mechanical switch (SW), a forced commutation branch, a flyback diode, and an energy absorption branch of metal oxide varistor (MOV) in parallel. Its topology is shown in Figure 1.

Under normal working conditions, the advantage of the small contact resistance of the mechanical switch is used to connect the circuit. In case of a fault short-circuit current, the working process is as follows:

(1) Firstly, the high-speed mechanical SW is triggered. After a phase of delay, the switch opens, and the air arc appears.
(2) After a short time of arcing, the thyristor (TH) of the forced commutation branch is triggered, and the pre-charged capacitor (C) discharges through the inductor to form a pulse current *i*_c_ opposite to the current *i*_SW_ direction of the switch, so as to gradually reduce the current *i*_SW_ until the current zero crossing is formed.
(3) Before the current *i*_SW_ crosses zero, the diode (D) cannot be turned on due to the clamping effect of arc voltage. The reverse pulse current does not flow through the diode until the arc is extinguished, providing the dielectric recovery time of zero voltage for the contact. In this process, the contact is still in motion, and the contact opening distance is increasing, which is conducive to the interruption of the circuit breaker.

**Figure 2:**
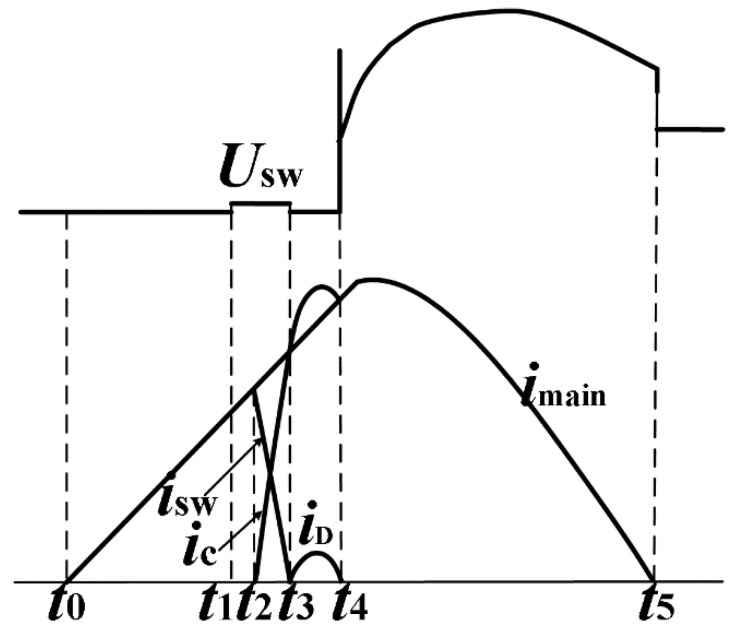
Schematic diagram of the voltage and current waveform during the working process of the circuit breaker.

Figure 2 shows a schematic diagram of the voltage and current waveform during the working process of the circuit breaker.

At *t*_1_, the mechanical switch is opened, and an arc occurs; at *t*_2_, the thyristor of the commutation branch is triggered; at *t*_3_, the commutation process ends and the arc is extinguished. Then, the commutation branch current passes through the diode branch to provide zero-voltage dielectric recovery time, which ends at *t*_4_, and overvoltage occurs.

## 3. Structure and Working Principle of the New Contact

**Figure 3:**
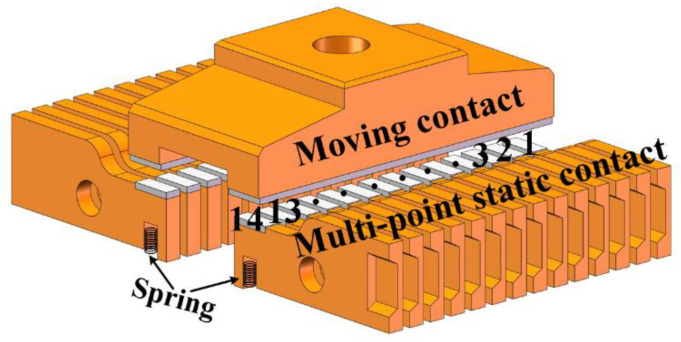
Structural diagram of the new contact.

In order to reduce the ablation effect of arc energy on the contact and improve the dielectric recovery speed after arcing, a new contact structure is proposed. Figure 3 is the structural diagram of the new contact, which is mainly composed of the moving contact, spring, and multi-point static contacts.

To improve the opening distance of the contact, the new contact adopts a bridge structure. The single-side static contact is composed of 14 finger contacts in parallel. A spring is installed under the finger of each static contact to obtain the contact overtravel during closing, which can ensure good contact between moving and static contacts without affecting the inherent time of the contact.

Under normal working conditions, due to the small difference in resistance among each finger contact, the current will flow evenly through each parallel contact. In case of a short-circuit current, the inductance of each parallel finger contact is used to delay the commutation time, ensuring that the current flows through each finger contact to form the arc mode of the parallel multi-column shape, which reduces the arc energy of each finger contact, so as to improve the dielectric recovery ability of the contact.

## 4. Experiment Validation

**Figure 4:**
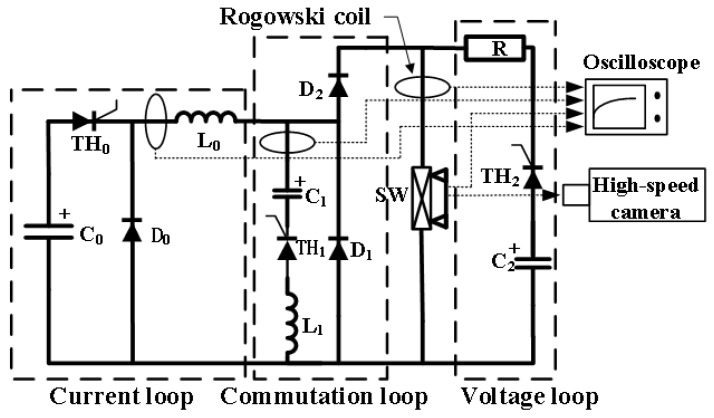
Schematic diagram of the experimental circuit.

**Figure 5:**
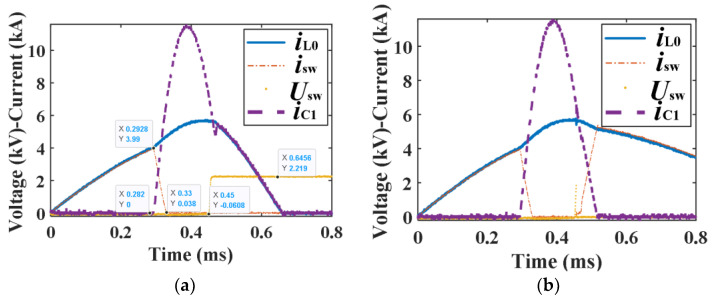
The typical working waveform of the contact. (**a**) Current and voltage waveform without breakdown. (**b**) Current and voltage waveform with breakdown.

**Figure 6:**
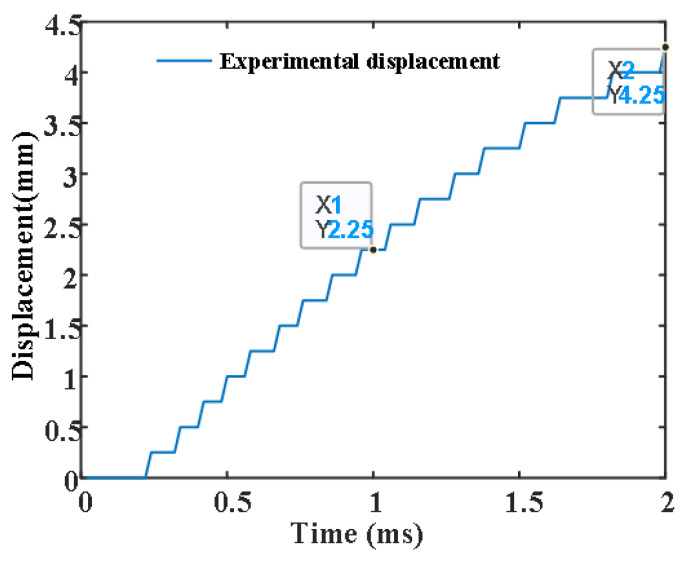
The displacement curve of the moving contact.

In order to verify the arc shape characteristics of the new contact in the separation process and study its post-arc dielectric recovery characteristics, the experimental circuit shown in Figure 4 is designed. The experimental circuit includes four parts: current loop, commutation loop, voltage loop, and signal acquisition. The current and voltage signals are collected by a Rogowski coil and voltage probe, respectively, and displayed on an oscilloscope; Figure 5 shows the typical current and voltage waveform of the new contact. The displacement curve and arc shape characteristics of the new contact are photographed using a high-speed camera; Figure 6 shows the displacement curve of the moving contact.

Based on the experimental circuit, the verification experiment of the arc shape characteristics and the dielectric recovery characteristic experiment of the contact after arcing have been carried out. The following mainly introduces the experimental process of the post-arc dielectric recovery experiment on the contact:

(1) At zero time, the thyristor TH_0_ of the current loop is triggered to generate a fault current. The fault current with different rising rates can be obtained by adjusting the inductance L_0_.
(2) When the current of the high-speed mechanical switch reaches the set value, the driving circuit of the mechanical switch is triggered. After a delay time, the high-speed mechanical switch is opened and the arc appears.
(3) After the switch is opened, the thyristor TH_1_ of the forced commutation loop is triggered after a delay time, and the pre-charged capacitor C_1_ discharges to form a reverse current. Since the arc voltage of the mechanical switch is applied to two ends of the diode D_1_, the diode D_1_ is cut off when the commutation loop is discharged, and the total current of the capacitor C_1_ flows through the mechanical switch. Different arc burning times can be obtained by controlling the triggered time of the thyristor TH_1_.
(4) When the current of capacitor C_1_ is greater than the current on the mechanical switch, the arc is extinguished and the mechanical switch branch is cut off. Thereafter, the current of capacitor C_1_ flows through diode D_1_, and the mechanical switch is in the dielectric recovery phase of zero voltage.
(5) During the dielectric recovery phase of zero voltage, the thyristor TH_2_ can be turned on to add the voltage of the capacitor C_2_ to the mechanical switch to test its breakdown voltage. Since the diode D_2_ is in the cut-off state, the voltage of the capacitor C_2_ will not affect the forced commutation loop and current loop; the resistance R of the voltage loop is used to limit the current of the voltage loop and avoid damage to the contact. The typical working process waveform is shown in Figure 5.

As can be seen from Figure 5a, the contact opens at 282 µs; after a short arcing time, the commutation starts at 292.8 µs, and the maximum current of the contact is 3990 A. The commutation process ends at 330 µs and enters into the dielectric recovery phase; after the commutation time of 120 µs, the withstand voltage 2219 V was applied at 450 µs, and the results showed that the contact is not broken down. Figure 5b shows that the contact is broken down when the voltage is changed to 2300 V; the critical breakdown voltage is considered to be 2250 V. By adjusting the triggered time of the commutation loop, the arc energy of the contact can be changed, and different dielectric recovery times and different breakdown voltages can be obtained by changing the triggered time of the voltage loop. Figure 6 shows the displacement curve of the moving contact, whose zero time is the triggered time of the high-speed mechanical switch, rather than the zero time when the fault current is triggered; the average speed of the contact is about 2 m/s.

## 5. Experimental Results and Discussion

### 5.1. Arc Shape Characteristics of the New Contact

**Figure 7:**
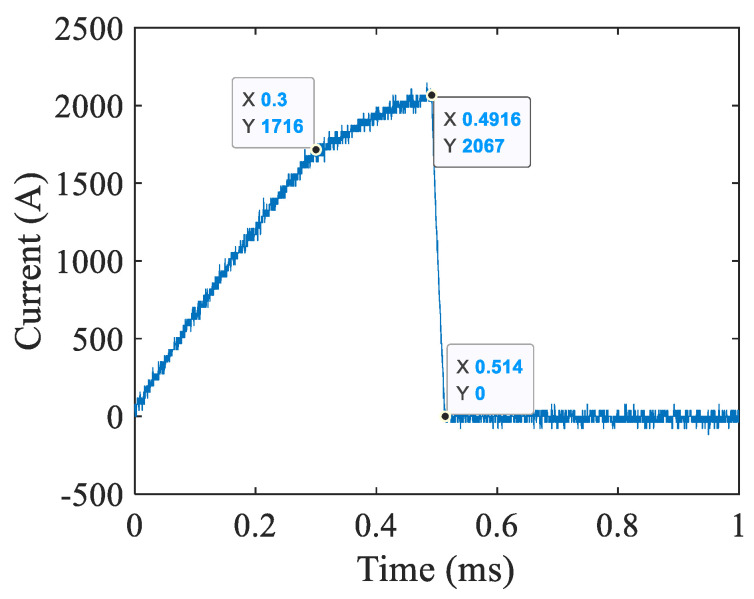
Current waveform of the new contact.

**Figure 8:**
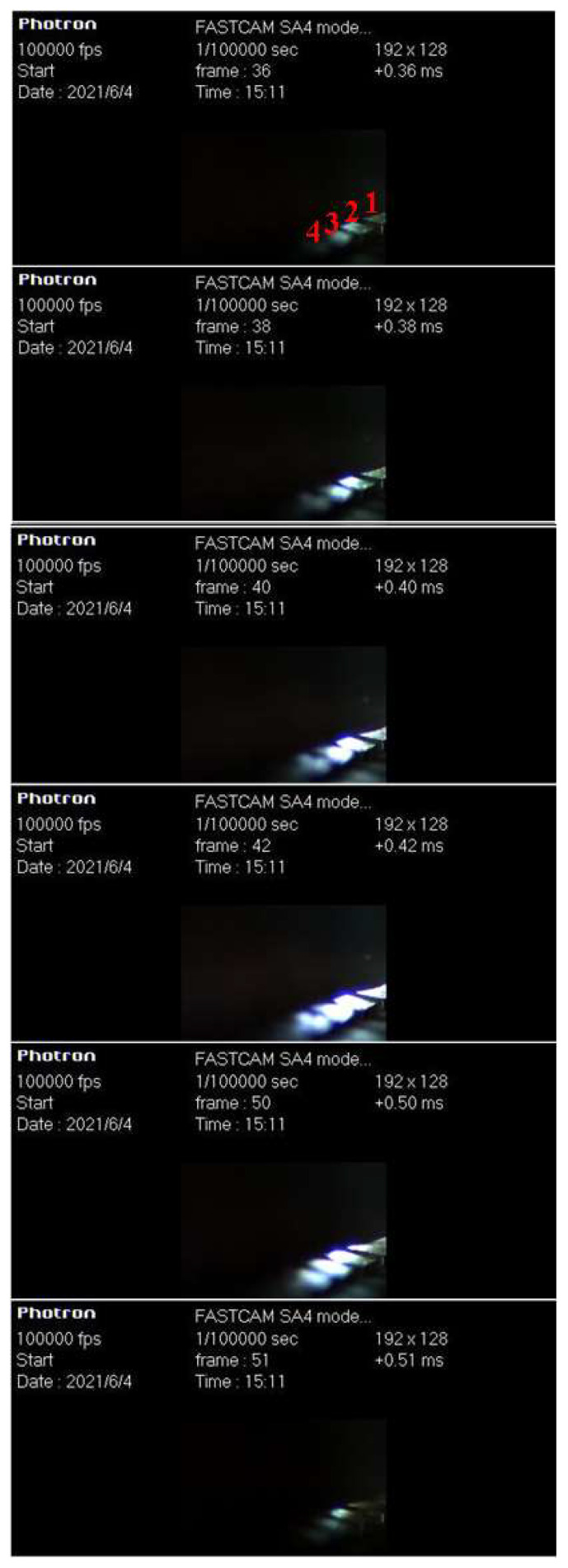
High-speed camera picture of the arc shape.

Firstly, the arc shape verification experiment of the new contact is carried out. The control sequence is as follows: trigger the current loop and high-speed mechanical switch at zero time and trigger the thyristor TH_1_ of the commutation loop at 492 µs. Since this experiment is mainly to observe the shape characteristics of arc, the high-voltage loop is not triggered. The arc shape of the new contact is photographed using a high-speed camera to verify that the parallel multi-point static contacts can produce multiple arc column burning phenomena. Figure 7 shows the current waveform of the new contact and Figure 8 shows the high-speed camera picture of arc shape.

It can be seen from the current waveform that the current rising rate suddenly changes at 300 µs. The reason for the sudden change is that the arc voltage of about 36 V is generated after the contact is opened, which changes the rising rate of current. At 491.6 µs, the thyristor TH_1_ of the commutation loop is triggered and starts the commutation process, and the current of the contact drops rapidly; at 514 µs, the current returns to zero and the arc extinguishes.

Figure 8 shows the high-speed imaging results of the arc shape. Due to the shooting angle, only the first four contact arcs can be captured. As can be seen from the figure:

(1) The new contact can generate multiple parallel arc columns when arcing.
(2) At 360~490 µs, the contact current increased, the heat generated by the arc has gradually accumulated, and the brightness of each finger contact has gradually increased with the increase in time; after 490 µs, the current decreases and the brightness of the contact decreases synchronously.
(3) The brightness of each finger contact is different at the same time. The brightness decreases from contact 2 to 4, indicating that the arc energy of each contact is inconsistent and that the current on each finger contact is different; due to the shooting angle, the arc of finger contact 1 is actually blocked, so its brightness is low.

### 5.2. Dielectric Recovery Characteristics of the New Contact

**Figure 9:**
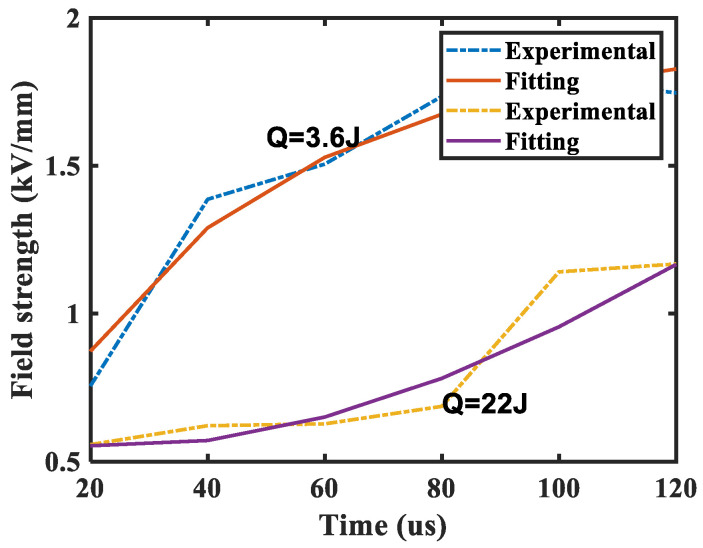
Dielectric recovery characteristic curves of the contact.

By changing the triggered time of commutation loop and voltage loop, the dielectric recovery characteristics of the new contact under different arc energy can be obtained. The current loop is triggered at zero time, the driving circuit of high-speed mechanical switch is triggered at 60 µs, and the commutation loop is triggered at 300 µs or 360 µs. After the arc is extinguished, the voltage loop is triggered at different times to obtain the critical breakdown voltage under different dielectric recovery times. As shown in Figure 9, the dielectric recovery characteristic curves are shown when the arc energy is 3.6 J and 22 J, respectively; here, arc energy represents the total arc energy. The field strength in Figure 9 is obtained by dividing the measured voltage by twice the measured contact displacement. The *x*-axis is the dielectric recovery time after the contact current being zero, not time zero at which the fault current is triggered.

It can be seen from the figure that:

(1) When the arc energy is 3.6 J, the critical breakdown field strength increases from 0.75 V/µm in the dielectric recovery time of 20 µs to 1.75 V/µm in the dielectric recovery time of 120 µs. The increasing rate of critical breakdown field strength with dielectric recovery time gradually tends to be flat.
(2) When the arc energy is 22 J, the critical breakdown field strength increases from 0.55 V/µm in the dielectric recovery time of 20 µs to 1.2 V/µm in the dielectric recovery timeof 120 µs. The increase rate of critical breakdown field strength with dielectric recovery time gradually tends to be increased.
(3) In the case of two different arc energies, the contact does not reach the cold breakdown field strength in the dielectric recovery time of 120 µs.
(4) In the dielectric recovery time of 120 µs, the greater the arc energy, the smaller the critical breakdown field strength and the worse the dielectric recovery ability after the same dielectric recovery time.

The dielectric recovery characteristics of air switches after arcing are related to many factors such as the contact structure, contact material, atmosphere, arc energy, contact opening distance, and so on. Under the conditions (arc energy 3.6~22 J, contact speed 2 m/s, and opening distance < 1 mm), only the effects of arc energy and contact opening distance on dielectric recovery characteristics are considered. The larger the contact opening distance, the faster the heat dissipation speed of the air in the contact gap, the smaller the time constant, and the faster the dielectric recovery speed will be; as the arc energy increases, the heat dissipation speed of the air in the contact gap decreases and the larger the time constant, the slower the dielectric recovery speed.

When the arc energy is 3.6 J, the influence of the distance relative to the arc energy on the recovery speed is small. Although the post-arc recovery time increases, the opening distance increases and the time constant decreases; there will be no situation where the dielectric recovery speed becomes faster and faster, so the post-arc dielectric recovery presents a gradually flat phenomenon.

When the arc energy is 22 J, the influence of the distance relative to the arc energy on the recovery speed is great. As the post-arc recovery time increases, the opening distance increases, and the time constant decreases, resulting in a situation where the recovery becomes faster and faster, so the post-arc dielectric recovery presents a gradually increasing phenomenon.

### 5.3. Analysis and Discussion of the Experimental Results

**Figure 10:**
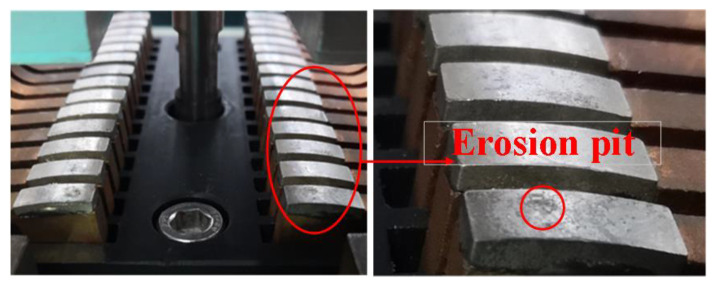
Picture of the contact surface after the dielectric recovery characteristic experiment.

After the experiment, the surface condition of the contact is photographed, as shown in Figure 10, which is a picture of the contact surface state after the dielectric recovery characteristic experiment.

It can be seen from the figure that except the contacts at two ends, other contacts have no obvious ablation marks. Once the contacts at two ends are broken down, the voltage of the contact will drop to arc voltage instantly, so the breakdown cannot occur on the other contacts, only on the contacts at two ends. The experimental results of dielectric recovery characteristics show that the arc energy has a great influence on the dielectric recovery ability. The higher the arc energy, the worse the dielectric recovery ability after the same dielectric recovery time. According to the high-speed camera shooting results of the arc shape characteristics of the new contact, the brightness of each contact is different at the same time, and the brightness gradually decreases from spots 2 to 4. Therefore, it can be seen that the current flowing through each contact also gradually decreases.

**Figure 11:**
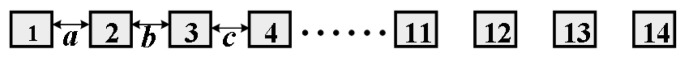
The definition of contact numbers and spacing parameters.

**Figure 12:**
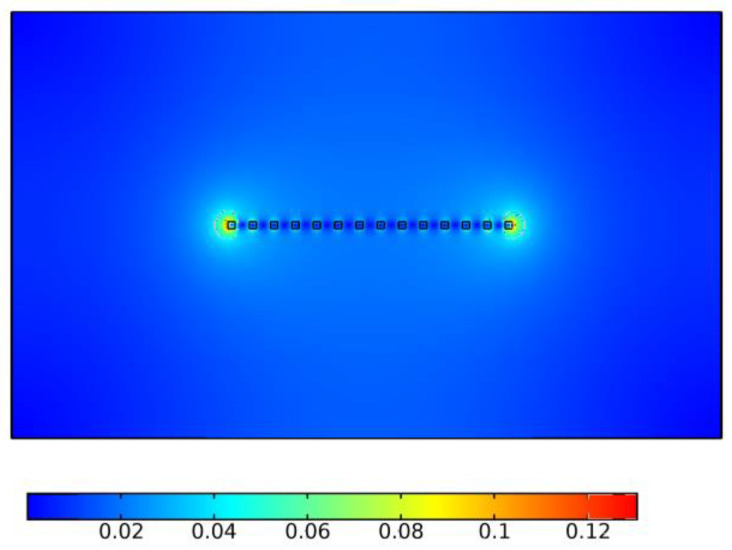
The distribution of magnetic flux density at 0.1 ms.

**Figure 13:**
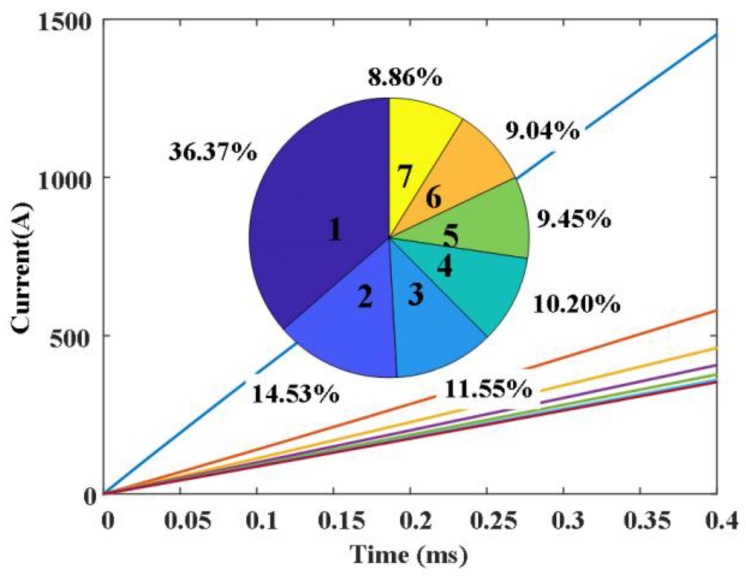
The proportional distribution of the currents in the contacts 1~7.

The finite element simulation model is established, and the current rising rate of 20 A/µs is applied to the contact. Figure 11 shows the definition of contact numbers and spacing parameters, Figure 12 shows the distribution of magnetic flux density at 0.1 ms, the unit is Tesla, and Figure 13 shows the proportional distribution of the currents of the 1~7 finger contacts (due to the symmetry of the structure, only the currents on the first seven contacts are displayed).

It can be seen from Figure 12 that due to the mutual repulsion of current between adjacent contacts, the magnetic field is ultimately concentrated at both ends of the contacts. It can be seen from Figure 13 that the current on each finger contact is different due to the magnetic flux coupling among each finger contact; the closer to the middle, the smaller the current, and the smaller the difference among the currents. The current flowing through contact 1 is 4 times that of contact 7 and 2.5 times that of contact 2; at the same time, the arc energy of each finger contact is also distributed in this proportion.

## 6. Optimization Design of the New Contact

According to the analysis above, the contact current will lead to weak breakdown points under the magnetic field coupling. Reducing the difference in current distribution is conducive to an improvement of the dielectric recovery ability after arcing. The electromagnetic field analysis model of the contact is established by using the finite element software. Because the length, width, and height of the contact have little influence on the difference in the current distribution, the influence of the distance among contacts on the contact current is analyzed.

**Figure 14:**
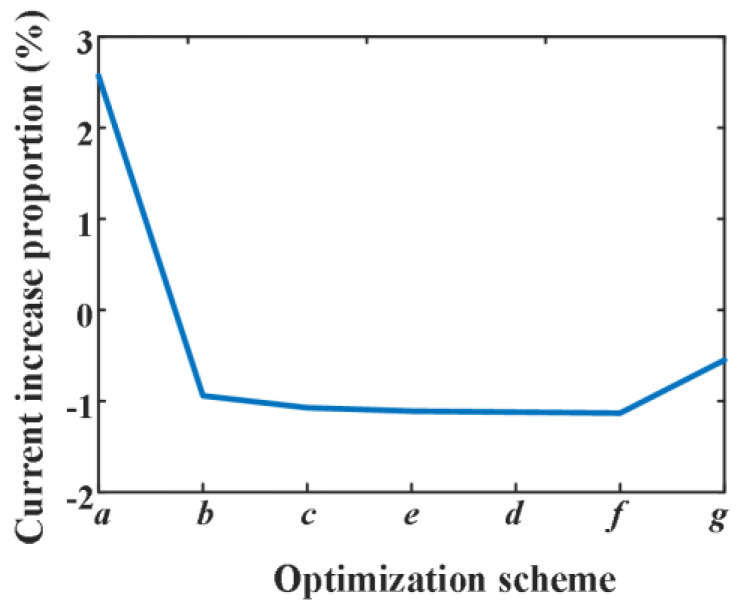
The influence of increasing parameters a~g on maximum current.

In the initial design, the distance between each finger contact is 2 mm, parameter a is the distance between contact 1 and 2, parameter b is the distance between contact 2 and 3, and so on. Because of the symmetrical structure of the contact, only parameters a~g need to be defined. By adjusting parameters a~g to 4 mm, respectively, the maximum contact current is obtained; due to the geometry of the contact, the maximum current always occurs at finger contact 1. Figure 14 shows the influence of increasing parameters a~g on maximum current.

In Figure 14, a~g represent different cases in which parameters a~g are changed, respectively; increasing the parameter a will increase the maximum current, whilst increasing other parameters will reduce the maximum current. The influence of the parameter a on the maximum current is greater than that of other parameters.

**Figure 15:**
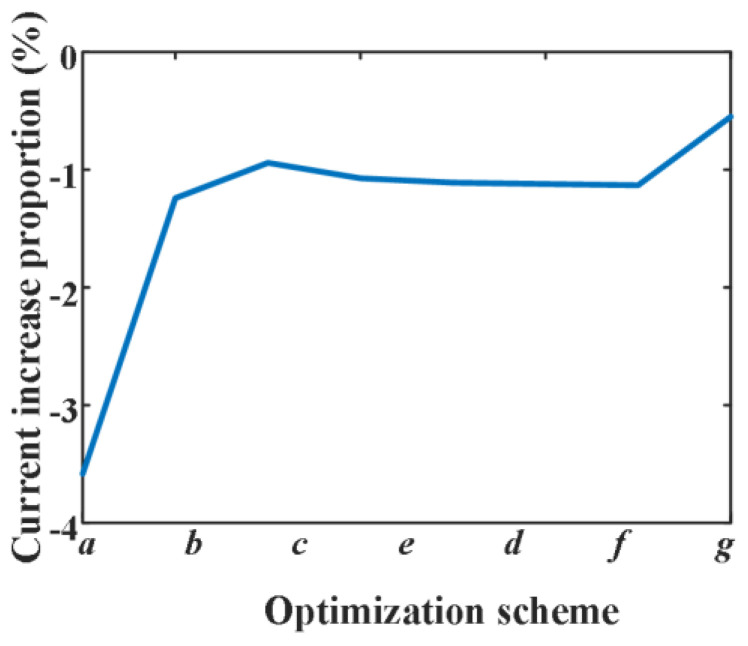
The influence of reducing parameters a~g on maximum current.

In order to reduce the maximum current, parameters a~g are, respectively, changed to 1 mm, and the simulation results are shown in Figure 15. It shows that the influence of the parameter a on the maximum current is greater than that of other parameters. Considering that the maximum current is affected by multiple parameters, a genetic algorithm (GA) is used for design optimization.

**Figure 16:**
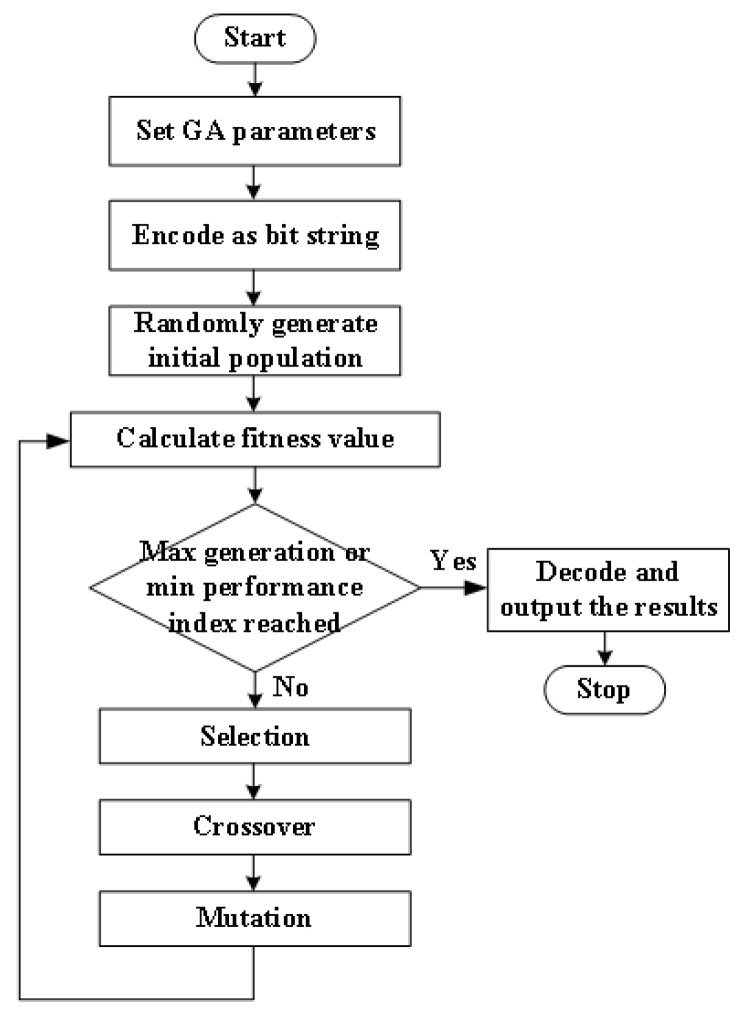
Schematic diagram of genetic algorithm calculation process.

GA is a search technique that mimics the mechanisms of natural selection. During GA computing, a population of artificial individuals is modified repeatedly based on biological evolution rules that converge toward a better solution to the problem being solved. At each step, individuals are selected at random from the current population to be parents. These individuals are used to produce children for the next generation. Based on biological basics, the fittest individuals survive and the least fit die. Through successful generations, the population evolves toward an optimal solution. Compared with other optimization techniques, GA is superior in avoiding local minima which is a common aspect of nonlinear systems. The general steps of the genetic algorithm are shown in Figure 16, and the main operational process is as follows:

(1) Encoding: The data in the solution space are used as a representation of genetic algorithms. The mapping from phenotype to genotype is called encoding. Before conducting a search, genetic algorithms first represent the solution data in the solution space as genotype string structure data in the genetic space, and different combinations of these string structure data form different points.
(2) Generation of initial population: Randomly generate N initial string structure data; each string structure data is called an individual and N individuals form a population. The genetic algorithm starts its iteration with these N string structures as the initial points. Set evolutionary algebra counters, set the maximum evolution algebra T, and randomly generate M individuals as the initial population.
(3) Fitness value evaluation detection: The fitness function indicates the superiority or inferiority of an individual or solution. The definition method of fitness function varies for different problems.
(4) Selection: Apply the selection operator to the population.
(5) Crossing: Apply the crossover operator to the population.
(6) Mutation: Apply the mutation operator to population.
(7) The population undergoes selection, crossover, and mutation operations to obtain the next population.
(8) Termination condition judgment: If the algebra is greater than the maximum evolutionary algebra, stop and output the optimal result; if not, skip back to step (2) and proceed with the iteration.

**Figure 17:**
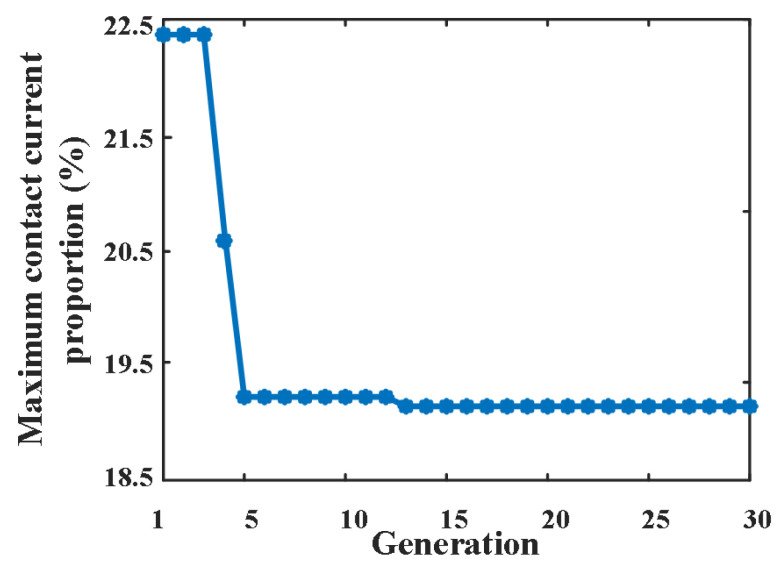
Variation curve of the proportion of maximum finger contact current to total current with generation.

Under the condition that the total distance among each contact is constant, parameters a~g are taken every 1 mm within the range of 1~5 mm. The fitness function is the proportion of maximum finger contact current to total current. The number of individuals per generation is 50. The crossover and mutation rates are 0.8 and 0.015, respectively. Figure 17 shows the variation curve of the proportion of maximum finger contact current to total current with generation. The optimal combination is a = 1, b = 4, c = 2, d = 4, e = 1, f = 1, g = 1. The comparison of parameter combinations before and after optimization is shown in Table 1.

In the case of individually increasing and decreasing each parameter, the increase and decrease in parameter a will lead to an increase and decrease in the maximum current, while the other parameters always decrease. However, when the sum of each parameter remains unchanged, the optimization results show a trend where parameters a, e, f, and g should be as small as possible, parameters b and d should be as large as possible, and parameter c should be an intermediate value.

The results show that the GA method can find an optimal combination, which can further reduce the maximum current compared with the equal distance scheme; the maximum current is only 19.07% of the total current, which greatly reduces the arc energy of the finger contact and improves the dielectric recovery ability of the contact.

## 7. Discussion

The new contact structure is suitable for low voltage and high-rated current situations. Compared to vacuum arc-extinguishing chambers, the new contact structure uses air as the arc-extinguishing dielectric, which has the characteristic of good heat dissipation performance; under the same contact quality, it can pass a larger current. Compared to the traditional butt contact, the new contact structure ensures the reliable contact of each finger contact through a static contact spring; this design does not affect the inherent time of the contact, and reduces the energy of a single ignition point and improves the dielectric recovery speed. In response to the current imbalance on various finger contacts of the new contact, the next step is to adopt a completely symmetrical structure to reduce the difference in current and further optimize the post-arc dielectric recovery environment. The new contact can be applied to DC fast protection scenarios such as microgrids, rail transit, all electric aircraft, distributed generation, and ship power systems, providing new ideas for the rapid protection of DC power systems.

## 8. Conclusions

To solve the problem of slow dielectric recovery speed caused by large arc energy when interrupting a high rising rate fault current in FDCCLCB, a new contact structure with the parallel multi-point static contacts is proposed. Through the experimental study on the dielectric recovery characteristics of the new contact, the following conclusions are obtained:

(1) The new contact is based on the principle that the parallel multi-point static contacts can produce the arc mode of multi-point arcing when the contact is opened, effectively reducing the arc energy of each finger contact and improving the dielectric recovery ability of the contact.
(2) When the arc energy is 3.6 J, the critical breakdown field strength increases from 0.75 V/µm in the dielectric recovery time of 20 µs to 1.75 V/µm in the dielectric recovery time of 120 µs.
(3) When the arc energy is 22 J, the critical breakdown field strength increases from 0.55 V/µm in the dielectric recovery time of 20 µs to 1.2 V/µm in the dielectric recovery time of 120 µs.
(4) The magnetic coupling among the finger contacts leads to a weak point of breakdown at two ends of the contact.

## Figures and Tables

**Table 1:** Comparison of parameter combinations before and after optimization.

Parameter	Before Optimization	After Optimization
a	2	1
b	2	4
c	2	2
d	2	4
e	2	1
f	2	1
g	2	1

## Data Availability

Data are available on request from the authors.
